# Use of Liquid-based Cytology and Cell Block Combined Technique for an Accurate Diagnosis of Oral Diffuse Large B Cell Lymphoma: A Case Report

**DOI:** 10.3390/diagnostics10100823

**Published:** 2020-10-14

**Authors:** Aya Yoshino, Shintaro Ishida, Shinsuke Nakamura, Ryosuke Kita, Mika Seto, Shinji Matsumoto, Morishige Takeshita, Seiji Kondo

**Affiliations:** 1 Department of Oral and Maxillofacial Surgery, Faculty of Medicine, Fukuoka University, 7-45-1 Nanakuma, Jonan-ku, Fukuoka 814-0180, Japan; ayayoshino@fukuoka-u.ac.jp (A.Y.); shintarou19910118@gmail.com (S.I.); nakamura0506@fukuoka-u.ac.jp (S.N.); rkita@fukuoka-u.ac.jp (R.K.); miichan@fukuoka-u.ac.jp (M.S.); 2 Department of Pathology, Faculty of Medicine, Fukuoka University, 7-45-1 Nanakuma, Jonan-ku, Fukuoka 814-0180, Japan; shinmts@fukuoka-u.ac.jp (S.M.); m-take@adm.fukuoka-u.ac.jp (M.T.)

**Keywords:** **Key words**: liquid-based cytology, cell block, diffuse large B cell lymphoma

## Abstract

Primary oral diffuse large B cell lymphoma (DLBCL) is rare and the differential diagnosis is difficult due to its low incidence and nonspecific symptoms, which resemble those of common oral diseases in the initial clinical setting. We aimed to discuss the value of making an accurate diagnosis using liquid-based cytology (LBC) and cell block (CB) for not only the morphological interpretation but also cytohistological assessment of oral DLBCL. LBC and CBs made from oral brushing materials were prepared on the first medical examination and a morphological analysis and immunohistochemical analysis of specific biomarkers were performed. The analysis of LBC preparations showed the presence of large-size lymphocytes with large irregular nuclei and prominent nucleoli, suggesting the existence of large B-cell lymphoma. A more detailed histological subclassification of the CB specimen was performed, which was classified as the activated B-cell (ABC) phenotype of DLBCL, by confirming the immunohistochemical expression of CD10−/ B-cell lymphoma 6 (BCL6)+/ multiple myeloma oncogene 1(MUM1)+, which is a significant risk factor in DLBCL. Our findings suggest that the combination of LBC and CB is a useful and informative tool for making an accurate molecular diagnosis of oral DLBCL in cases in which lymphomas are clinically suspected.

## 1. Introduction

Lymphomas are malignant neoplasms of the lymphocyte cell lines that affect the lymph nodes, spleen and other nonhemopoietic tissues [1]. They are classified as either Hodgkin’s or non-Hodgkin’s Lymphoma (NHL), and either B-cell or T-cell according to their cell of origin [1]. Extranodal lymphomas tend to most commonly occur in the gastrointestinal tract, followed by the head and neck region [2,3]. Mainly formed from the Waldeyer’s ring, the hard palate and the maxillary vestibule appears to be frequently involved [2]. NHLs of the oral cavity account for only 3–5% of reported lymphomas, and the most frequent type of oral NHL is diffuse large B cell lymphoma (DLBCL) [3].

DLBCL is an aggressive, neoplasm of large lymphoid cells that exhibits rapid monthly growth. This heterogenous neoplasm has variable cytomorphologic, immunophenotypic, and genetic features [1]. Since the response to treatment regimens and prognosis of the molecular subtypes differ, for example, the germinal center B-cell-like (GCB) subtype tends to exhibit a better prognosis than the activated B-cell-like (ABC) subtype [4], the best method for managing lymphoma is determined according to subtypes classified at the gene expression level based on comprehensive genetic analyses, such as flow cytometry, fluorescence in situ hybridization, and cDNA microarray techniques. This facilitates molecular-targeted and personalized treatment planning without delay [1,2,3,4]. Likewise, it is also important to determine the cell-of-origin as soon as possible in case of DLBCL arising in the oral cavity. However, these molecular diagnostic approaches may not be used in routine clinical practice because of high costs and the need for specific technology. Moreover, the clinical situation at the time of the initial diagnosis of oral DLBCL is confusing, since oral DLBCL shows atypical symptoms, which present as a nonspecific enlarging swelling, ulceration and discomfort in the region of involvement, which mimic inflammatory odontogenic or periodontal diseases [5]. Thus, immunohistochemistry-based algorithms with subsequent excisional biopsy was initially used as an easy and reliable method for determining the molecular subtype of DLBCL, including DLBCL arising in the oral cavity.

Exfoliative cytology has various advantages. This noninvasive, inexpensive, and painless diagnostic modality has been applied in many fields of medicine, including gynecology [6]. Thus, this method was initially applied in the diagnosis of disease of the oral cavity instead of excisional biopsy. However, it has been considered to show minimal diagnostic value due to high false-negative rates derived from mechanical and stain artifacts with block sampling due to the thick keratin layer and/or destruction of the three-dimensional presentation [7]. Liquid-based cytology (LBC) is a thin-layer slide preparation procedure that was developed to overcome the cell crowding and contamination associated with conventional exfoliative cytology [7,8]. A cellular suspension is obtained using a cytobrush, and several slides can be prepared and examined within a few days. Moreover, the residual specimens after LBC processing can be used for cell block (CB) preparation, which can be used for ancillary analyses. Of note, the main advantage of CB preparation is the potential to produce several sections for immunohistochemistry and molecular diagnostics, that is, the application of cytohistology in the diagnosis of oral DLBCL, with a minimal excisional biopsy [9,10].

In the present study, we discuss the usefulness of the combination of LBC and CB using oral brushing materials to make the initial diagnostic interpretation of oral DLBCL without time-consuming procedures.

## 2. Materials and Methods

### 2.1. Sample Collection and Liquid-Based Cytology

Oral specimens were harvested from suspicious lesions, the right upper posterior tooth extraction socket by gentle scraping with a cytobrush. After smearing the specimens onto a slide for conventional air-dried May–Grünwald–Giemsa staining, the cytobrush was directly inserted into a single vial containing a liquid-based fixation medium, CytoRich™Red (TriPath, Burlington, NC, USA) followed by LBC processing using a BD SurePath™ system (TriPath). Cells were transferred to the slide for LBC using air pressure for adherence. Slides were fixed in 95% ethanol for 24 h, stained using the Papanicolaou procedure and examined under a light microscope (OLYMPUS BX53, Olympus Life Science, Japan).

### 2.2. Preparation of Cell Blocks

Cell blocks (CBs) were prepared from the residual BD SurePath sample described above by the formalin superposition method. Briefly, the residual sample from the LBC preparation was centrifuged at 3000 rpm for 2 min. After decanting the supernatant, the resulting pellet was fixed and overlaid with 10% formalin-buffered solution. Pellets were placed in a cassette then embedded in paraffin wax and trimmed in 3 µm-thick sections for hematoxylin and eosin (HE) staining and immunocytochemistry. In this way, the CB analysis offered the advantage of developing multiple sections of paraffin-embedded cells, the ability to observe structural patterns, and enabled the performance of IHC.

### 2.3. Immunocytochemical Staining

Immunohistochemistry was performed according to a standard protocol with antibodies against Ki67, BCL2, BCL6, MUM1, CD10, CD20, CD79a, CD3, CD25, CD30, CD56, cyclin D1 and MYC. Slides made from cell blocks and/or tissue excisional biopsy were treated in the DAKO Omnis automated slide preparation system (Dako, Carpinteria, CA, USA and Glostrup, Denmark) using an EnVision FLEX kit (Dako). The primary antibodies, anti-BCL2 (124, 1:500; Agilent Technologies, Santa Clara, CA, USA), anti-BCL6 (PG-B6p, prediluted from manufacturer; Agilent Technologies), anti-MUM1 (MUM1 p, 1:100; Agilent Technologies), anti-CD3 (PS1, prediluted from manufacturer; Leica Biosystems, Newcastle, UK), anti-CD10 (56C6, prediluted from manufacturer; Agilent Technologies), anti-CD20 (L26, prediluted from manufacturer; Agilent Technologies), anti-CD79a (SP18, prediluted from manufacturer; Nichirei Biosciences, Tokyo, Japan), anti-CD25 (4C9, 1:200; Leica Biosystems, Baffalo Grove, IL,USA), anti-CD30 (Ber-H2, 1:50; Agilent Technologies), anti-CD56 (123C3, prediluted from manufacturer; Agilent Technologies), anti-cyclin D1(SP4, prediluted from manufacturer; Nichirei Biosciences), anti-MYC (Y69, 1:200; abcam, Burlingame, CA, USA) and anti-Ki67 (MIB-1, prediluted from manufacturer; Agilent Technologies), were diluted in 1% bovine serum albumin and 0.1% Na_3_N on PBS (pH 7.4), followed by overnight incubation (18 h) at 4 °C. Epstein–Barr virus encoded RNA (EBER) in situ hybridization was performed using an immunostainer (Leica BOND MAX, Leica Biosystems).

Based on the immunophenotypical expression, the two most common subgroups—germinal center B-cell like (GCB) and activated B-cell like (ABC)—were classified using antibodies against CD10, BCL6 and MUM1 [1,4]. If both CD10 and BCL6 are positive or if CD10 alone is positive the tumor can be classified into the GCB subgroup. If CD10 is negative and BCL6 is positive, the expression of MUM1 determines the subgroup. If MUM1 is negative, the tumor is classified into the GCB subgroup, whereas if MUM1 is positive, the tumor is classified into the ABC subgroup.

### 2.4. Case Description

**Figure 1:**
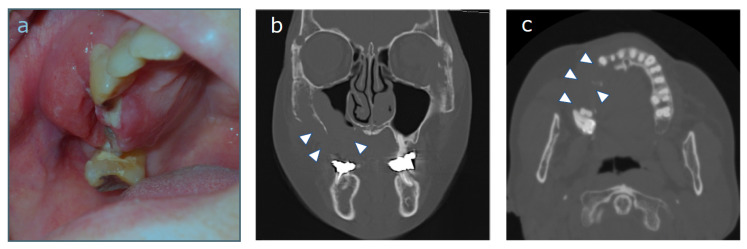
(**a**) An intraoral view at the first examination. A localized, well-defined swelling of size 4 cm × 5 cm in its greatest dimensions was obliterating the buccal and palatal vestibules in the region of the unhealed extraction sockets of 14, 15 and 16. Coronal (**b**) and axial (**c**) computed tomography imaging showed the osteolytic focus in the maxilla (white arrowhead).

**Figure 2:**
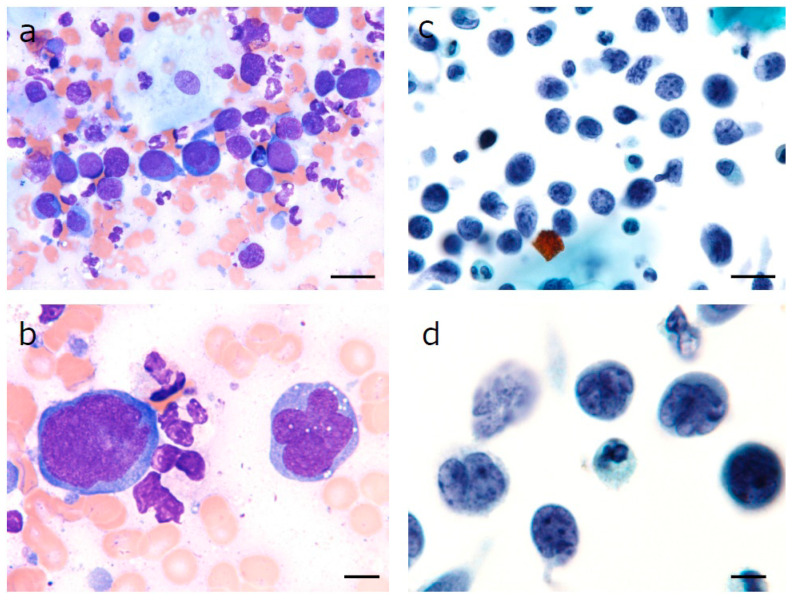
Microscopic view of oral specimens harvested from suspicious lesions. Large atypical lymphocytes with an irregular nuclear shape (Giemsa staining, (**a**) magnification 40×, bar = 20 µm (**b**) magnification 100×, bar = 5 µm). In the liquid-based cytology (LBC) specimen, (**c**) large atypical lymphocytes with a high nuclear/cytoplasmic ratio were recognized with prominent nucleoli (Papanicolou staining, magnification 40×, bar = 20 µm); (**d**) The nuclear strips implied nuclear fragility (Papanicolou staining, magnification 100×, bar = 5 µm).

**Figure 3:**
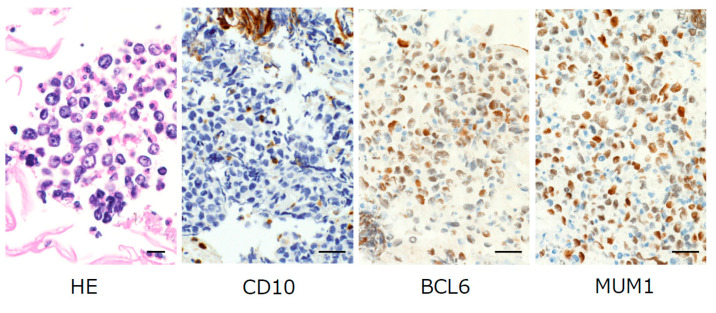
Hematoxylin–eosin (HE) staining of cell blocks showed large lymphocytes (magnification 40×, bar = 20 µm). Immunostaining of cell blocks (magnification 20×, bar = 50 µm) showed nuclear positivity for B-cell lymphoma 6 (BCL6), and multiple myeloma oncogene 1 (MUM1). The specimen was CD10-negative. We confirmed positive staining of CD79a, CD20, BCL2, and Ki67 in large lymphocytes that were identified by HE staining. Negative staining of CD3, CD25, CD30, and CD56 was not shown.

A 67-year-old woman was referred to our department with a chief complaint of swelling in her right cheek. She had unhealed tooth extraction sockets from right upper posterior tooth extraction that had been performed approximately one month previously. A laboratory examination revealed an abnormal GTP level and a slightly elevated serum CRP level. The patient’s relevant history included treatment for colorectal cancer. An intra-oral examination revealed a localized, well-defined swelling of 4 cm × 5 cm in its greatest dimensions that obliterated the buccal and palatal vestibules in the region of the unhealed extraction sockets of 14, 15 and 16 (Figure 1a). Computed tomography (CT) of the head and neck showed destruction of the right maxillary alveolus extending antero-posteriorly to the zygomatic bone and enhancing masses in the right paranasal sinuses associated with the destruction of the adjacent bony structures (Figure 1b,c). Based on the clinical suspicion of malignancy, the combination of the LBC and CB techniques for the analysis of oral brushing materials was performed at the time of the first medical examination. Giemsa-stained slides revealed large lymphocytes with an irregular nuclear shape (Figure 2a, 40× magnification). A slightly basophilic cytoplasm without azurophilic granules was well recognized (Figure 2b 100× magnification). Papanicolaou-stained slides revealed a cleft, large irregular nuclei containing hyper and granular chromatin, which were observed at both (c) 40× and (d) 100× magnification, which was cytomorphologically suggestive of DLBCL (Figure 2c,d). For a more accurate diagnosis, we performed IHC using the CB technique with the formalin superposition method. HE staining showed large irregular nuclei with coarse, granular, dispersed chromatin and large nucleoli. IHC showed atypical lymphoid cells that were positive for MUM1, BCL6, and BCL2, with intense staining of CD79a, CD20, and Ki67, whereas the specimens were negative for CD10, CD3, CD25, CD30, and CD56 (Figure 3). Finally, our case was classified as the ABC phenotype after confirming the expression of CD10−/ BCL6+/ MUM1+.

**Figure 4:**
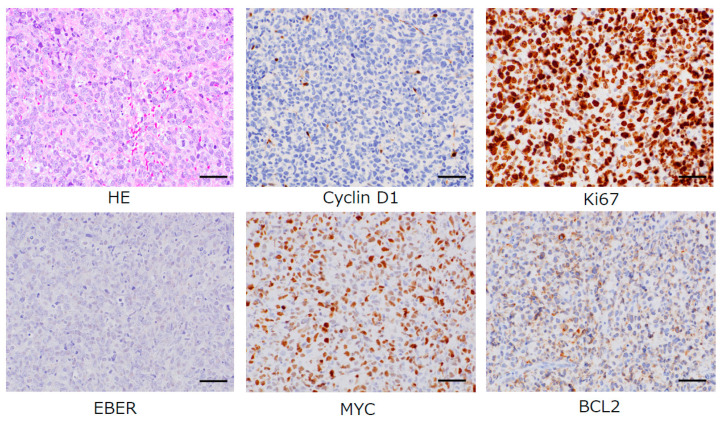
Diffuse infiltration of neoplastic large B cells (HE, magnification 200×, bar = 100 µm) with negative cyclin D1 immunostaining (magnification 200×, bar = 100 µm) was recognized. Immunostaining of Ki67 (90% of neoplastic cells, magnification 200×, bar = 100 µm), BCL2 (40% of neoplastic cells, magnification 200×, bar = 100 µm), and MYC (60% of neoplastic cells, magnification 200×, bar = 100 µm) showed positive reaction for these antibodies. Epstein–Barr virus (EBV)-associated diffuse large B cell lymphoma (DLBCL) was not recognized, as Epstein–Barr virus encoded RNA (EBER) in situ hybridization was negative (magnification 200×, bar = 10 µm). The morphologic, immunophenotypic, and molecular/genetic features allowed for the identification of a prognostically important subtype: activated B-cell (ABC)-DLBCL with the co-expression of MYC/BCL2.

At one week before re-examination, a minimal excisional biopsy specimen retrieved non-formalin-fixed fresh tissue from the palatal aspect of the posterior right maxilla in which cell surface antigen and specific chromosomal/genetic abnormalities could be evaluated by comprehensive genetic analyses, and the ABC phenotype of DLBCL was re-confirmed along with negative cyclinD1, negative EBER, following the detection of the double-expression of the MYC and BCL2 proteins, an important risk factor [1], by comprehensive genetic analyses (Figure 4). The patient was referred to the hematology department where she underwent treatment without delay.

## 3. Discussion

In the clinical setting, exfoliative cytology can be applied as a first line diagnostic modality due to its advantages of being simple, cost-effective and minimally invasive [7]. However, this method is limited in terms of its sensitivity. We therefore overcame the limits of exfoliative cytology using the combination of LBC and CB as a valuable first-line medical examination for oral diseases. CB and IHC helped us to establish an accurate diagnosis of oral DLBCL due to its potential to produce several sections for immunohistochemistry, although the final diagnosis of DLBCL was confirmed by tissue excisional biopsy and subsequent IHC and in situ hybridization studies. In fact, in our case DLBCL could be predicted by LBC from oral brushing materials, with further classification into the ABC subtype of DLBCL based on an immunohistochemical analysis of the CB to detect three markers: CD10, BCL6 and MUM1. Cytohistological analyses using CB in addition to IHC for conventional exfoliative cytology have the potential to fill the ‘‘screening gap’’, by achieving the accurate molecular diagnosis of oral diseases as well as for cancer and precancer screening, and can enable an accurate diagnosis, with similar accuracy to tissue biopsy, which can better evaluate the architectural patterns. That is, the CB procedure is considered to represent a minimally invasive brush biopsy procedure [10]. Thus, the combination of LBC and CB has the potential to minimize the need for tissue excisional biopsy for diagnostic confirmation. Since these series of analyses could be performed without time-consuming procedures, the next examination and molecular-targeted treatment could be carried out without delay. It is crucial to note that early detection and an accurate diagnosis before personalized treatment can result in a complete cure and better long-term survival in DLBCL patients.

In conclusion, accurate molecular diagnosis of oral DLBCL could be supported by integrating CB and IHC into LBC using oral brushing materials to facilitate appropriate treatment planning in the clinical setting. The combination of LBC and CB has attracted increasing attention in the era of personalized medicine.
